# Pontocerebellar Hypoplasia Type 1D: A Case Report and Comprehensive Literature Review

**DOI:** 10.3390/jcm11154335

**Published:** 2022-07-26

**Authors:** Ivana Dabaj, Adnan Hassani, Lydie Burglen, Leila Qebibo, Anne-Marie Guerrot, Stéphane Marret, Abdellah Tebani, Soumeya Bekri

**Affiliations:** 1 Department of Neonatalogy, Pediatric Intensive Care and Neuropediatrics, CHU de Rouen, F-76000 Rouen, France; stephane.marret@chu-rouen.fr; 2 Department of Metabolic Biochemistry, Normandie University, UNIROUEN, CHUROUEN, INSERM U1245, F-76000 Rouen, France; abdellah.tebani@chu-rouen.fr (A.T.); soumeya.bekri@chu-rouen.fr (S.B.); 3 Department of Radiology, CHUROUEN, F-76000 Rouen, France; adnan.hassani@chu-rouen.fr; 4 Centre de Référence “Malformations et Maladies Congénitales du Cervelet”, APHP, Sorbonne Université, F-75012 Paris, France; lydie.burglen@aphp.fr (L.B.); leila.qebibo@aphp.fr (L.Q.); 5 Département de Génétique, Hôpital Armand Trousseau, APHP, Sorbonne Université, F-75012 Paris, France; 6 Developmental Brain Disorders Laboratory, Imagine Institute, INSERM UMR 1163, F-75015 Paris, France; 7 Department of Genetics, CHUROUEN, F-76000 Rouen, France; anne-marie.guerrot@chu-rouen.fr; 8 Department of Metabolic Biochemistry, CHUROUEN, F-76000 Rouen, France

**Keywords:** pontocerebellar hypoplasia, EXOSC9, cerebellar atrophy, spinal motor neuronopathy, motor neuron disease

## Abstract

Pontocerebellar hypoplasia (PCH) is an autosomal recessive, neurodegenerative disorder with multiple subtypes leading to severe neurodevelopmental disabilities. PCH type 1 D is linked to alterations in the *EXOSC9* gene. EXOSC9 is a component of the RNA exosome, an evolutionarily conserved ribonuclease complex essential for RNA degradation and processing. The clinical phenotype is characterized by cerebellar and pontine hypoplasia associated with motor neuronopathy. To date, nine patients have been reported in the literature with PCH1D. We report the case of an infant with PCH type 1D due to two variants in the *EXOCS9* gene (NM_001034194.1: c.41T>C-p.Leu14Pro) and a novel variant (c.643C>T-p.Arg212*). This report thoroughly reviews the literature PCH1D and highlights the crucial role of the exosome in cellular homeostasis.

## 1. Introduction

Pontocerebellar hypoplasias (PCH) are a group of genetically and clinically heterogeneous disorders characterized by severe neurodevelopmental delay, neurodegeneration and specific neurologic symptoms depending on the subtype [1,2,3]. Radiologically, all patients have cerebellar and pontine hypoplasia or atrophy as well as variable cerebral involvement [1,3]. There are 17 types of PCH in OMIM (https://www.omim.org/, accessed on 15 May 2022). Type 1 and 2 PCH were the first types described and currently they include six subtypes each (A, B, C, D, E, F). Concerning the molecular findings and pathophysiology, the RNA exosome is an evolutionarily conserved ribonuclease complex essential in RNA degradation and processing [4,5,6,7]. This complex is composed of nine subunits, EXOSC1-9: EXOSC1-3 constitute the cap and EXOSC4-9 the core. The exosome is associated with nuclear or cytoplasmic cofactors to target several RNA types such as pre-mRNA, m-RNA, and t-RNA [5,6,8,9]. Variants in *EXOCS3, EXCOS8* and *EXOCS9* genes have been associated with pontocerebellar hypoplasia (PCH) and spinal motor neuron dysfunction (PCH type 1 B, OMIM #614678; PCH type 1C, OMIM #616081; and PCH type 1D, OMIM #618065, respectively). In addition, central nervous system demyelination has been described in patients with *EXOSC8* gene alteration [4,5,8,10,11,12,13]. Variants in the *EXOSC2* gene cause a different phenotype with a complex syndrome presenting with mild developmental delay, dysmorphia, ophthalmological abnormalities (myopia, early onset retinitis pigmentosa), progressive sensorineural hearing loss, hypothyroidism, and premature ageing [14,15]. Furthermore, variants in the gene coding for RBM7, a protein interacting with the exosome, leads to motor neuron disease with a spinal muscular atrophy-like phenotype [16].

In this report, we describe the case of a boy presenting with severe developmental delay, motor neuronopathy, cerebellar atrophy and pontine hypoplasia. The clinical phenotype and workup are consistent with PCH type 1D due to variants in the *EXOSC9* gene.

## 2. Patient and Methods

### 2.1. Case Description

A 21-month-old boy, born at 35 weeks + 6 days, by normal vaginal delivery presented with neonatal distress, APGAR 6, 10 and 10 at 1, 5 and 10 min respectively. Umbilical cord blood gas showed a pH of 7.3. At birth he had a weight of 2455 g, height of 44 cm, head circumference of 32 cm, and was fed by an NG-tube due to feeding difficulties. This newborn required early phototherapy for physiologic neonatal jaundice. There were no hematological, hepatic, or infectious disorders. He was then transferred to our neonatal intensive care unit at 11 days post-natal for management of hypotonia. On physical exam he had generalized severe hypotonia, limited spontaneous and antigravity movements, occasional eye contact, intermittent nystagmus, hypomimia, weak cry, deep tendon reflexes 1+, poor suckling reflex, no rooting reflex, and normal cardiorespiratory and abdominal exams.

### 2.2. Brain Magnetic Resonance Imaging (MRI)

A cerebral MRI (1.5 T) was performed using T1 and T2 weighted imaging, allowing anatomical information and brain maturation assessment.

### 2.3. Neurophysiological Investigations

The patient underwent electromyography (ENMG) study using a portable Synergy machine. Before each study the temperature was checked, and cool limbs (<32 °C) were warmed. Electroencephalogarphic (EEG) study was done using Nicolet machine with 16 electrodes using a 10/20 system adapted for neonates.

### 2.4. Biochemical Investigations

For urinary organic acids, urines samples were subjected to derivatization with N,O-bis(trimethylsilyl)trifluoroacetamide and trimethylchlorosilane. Derivatized samples were injected into a Shimadzu QP-2010 Plus GC-MS operating in split mode. The metabolites were analyzed as trimethylsilyl compounds. Heptadecanoic acid was used as an internal standard. A Blood Acylcarnitine Profile on a dried blood spot was generated using butylation derivatization (ChromSystems**^®^**, Munich, Germany) and measured by MS/MS on a 4000 QTRAP (Sciex**^®^**, Concord, ON, Canada). The acylcarnitine butylated esters were acquired by precursor ion scanning of 85 *m/z* in positive ion mode.

### 2.5. Molecular Analysis

The genomic DNA of the patient was analyzed using a targeted “Congenital cerebellar anomalies and childhood movement disorders” gene panel (277 genes). This panel includes 30 genes involved in PCH, including *EXOSC9*. Samples were prepared with the SeqCap EZ Choice preparation kit (Roche) and sequenced on an Illumina MiSeq sequencer using 2 × 150 bp sequencing kits. The Basespace cloud computing platform (with BWA 2.1 and GATK Unified Genotyper 1.6) and the Variant Studio v.3.0 software provided by Illumina were used. *EXOSC9* variants were confirmed by Sanger sequencing in the index case and both parents. Pathogenicity of variants was ascertained according to the ACMG (American College of Medical Genetics) criteria.

## 3. Results

### 3.1. Brain MRI

**Figure 1:**
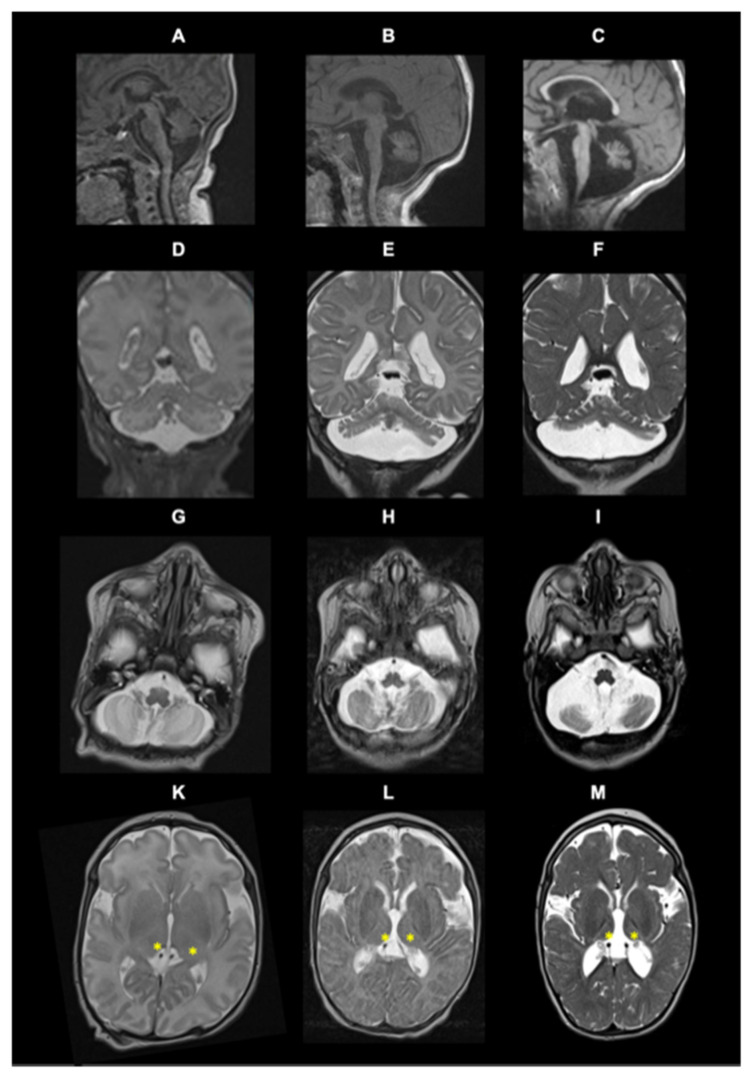
Brain MRI of the index case at the age of 2 weeks (**A**,**D**,**G**,**K**), 3 months (**B**,**E**,**H**,**L**), and 20 months (**C**,**F**,**I**,**M**). Description from first to last raw: sagittal T1 (**A**,**B**,**C**); coronal T2 (**D**,**E**,**F**); and axial T2 (**G**,**K**,**H**,**L**,**I**,**M**).

At 2 weeks of life, the MRI images showed discrete cerebellar hypoplasia (including vermis) (Figure 1A,D,G) and delayed myelination with the absence of myelin in the posterior limb of the internal capsule normal pons (Figure 1A) and thalami (Figure 1K). MRI controlled at the age of 3 months and 20 months showed progressive pontine and vermis atrophy (Figure 1B,C) as well cerebellar hemisphere atrophy (Figure 1G,K), persistent delayed myelination, and progressive thalami atrophy (Figure 1K,L,M).

### 3.2. Molecular, Biochemical and Neurophysiological Findings

The workup conducted initially showed no abnormalities on transfontanellar ultrasound, arterial blood gas, TORCH serology, auditory evoked potential, CBC, CK, lactate, pyruvate, ammonia, acyl carnitine profile, long chain fatty acid, urinary organic acid chromatography, and plasmatic amino acid chromatography.

Amplitude integrated EEG conducted at 2 weeks of life for 3 days showed some discontinuous background activity but no abnormal epileptic discharge. ENMG at 3 months showed chronic motor axonal neuropathy with bulbar involvement. CGH array and sequencing for lysosomal disorders were negative.

The targeted NGS panel for cerebellar abnormalities revealed compound heterozygote variants in the *EXOSC9* gene: one previously described variant, NM_001034194.1: c.41T>C (p.Leu14Pro) [8] and a novel variant, c.643C>T (p.Arg212*). This new variant in the *EXOSC9* gene is present in a minor allelic frequency in GnomAD. It leads to the interruption of the reading frame due to a premature codon stop. The presence of this variant in addition to the other variant p.(Leu14Pro) is responsible for the pontocerebellar hypoplasia in the index case. Thus, our patient has PCH type 1D.

## 4. Discussion

Pontocerebellar hypoplasia (PCH) is an autosomal recessive, neurodegenerative disorder leading to a severe neurodevelopmental disease [1,2]. The phenotype has been expanded since the first description [17] to currently include 13 subtypes with almost 20 causative genes [3] and variable clinical as well as radiological features, all of which have concurrent hypoplasia or atrophy of the cerebellum and the pons [1,3]. The first two subtypes described were PCH1 and PCH2 with distinct neuropathological findings (absence of motoneuron disease in PCH2 while progressive motor neuronopathy is present in PCH1) [1,6]. As a consequence, muscular atrophy and hypotonia are described in PCH1 [2] while dyskinesia and spasticity are the main features in PCH2 [2,18]. Later, the classification was based on radiological, biochemical, and genetic findings in addition to the clinical features [1,2,3]. All patients have severe developmental delay (rarely mild) and present during the first months of life with visual disorder, seizures, and feeding problems in addition to the psychomotor delay [2]. PCH1 is a lethal disorder, classified as type 1A with variants in the *VRK1* gene [1,19,20,21], type 1B with *EXOS3* variants [1,2,13,22], type 1C with *EXOSC8* variants [1,10], type 1D with *EXOSC9* [4,8] and type 1E with *SLC25A46* variants [1,23,24]. PCH1 due to *EXOSC9* dysfunction was first described by Burns et al. [8].

**Figure 2:**
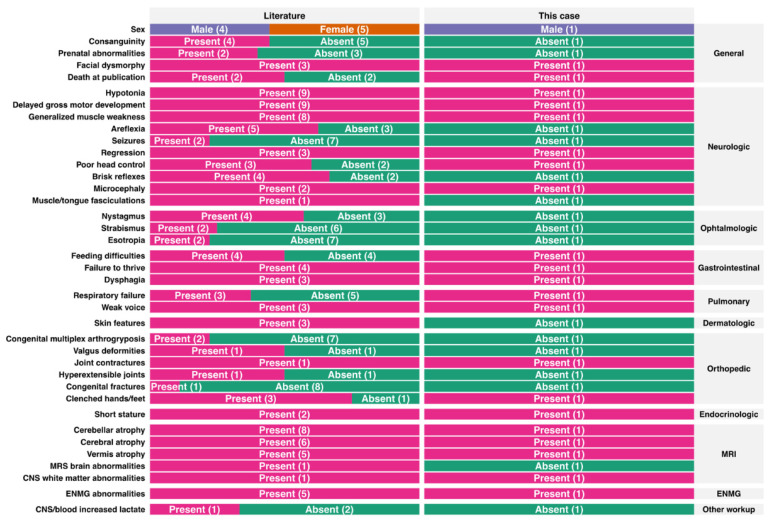
Clinical presentation overview.

We report the case of a boy who presents with PCH1 features (Table 1). Genetic analysis allowed the identification of two variants in the *EXOCS9* gene which correspond to the PCH type 1D phenotype. The first variant found has been previously described (NM_001034194.1: c.41T>C-p.Leu14Pro) [8] and the other is novel nonsense pathogenic variant (c.643C>T-p.Arg212*). To date, nine patients were reported in the literature with the PCH1D phenotype. In all the patients described, including the present case, 20 mutated alleles were identified. The c.41T>C-p.Leu14Pro variant is the most common (in 60% of the cases); the others are c.239 T>G-p.Leu80Arg (10%), c.484 DupA-p.Arg162Lysfs*3 (10%), c.151G>C-p.Gly51Arg (10%), c.481 C>T-p.Arg161* (5%) and c.643C>T-p.Arg212* (5%) (Supplementary Table S1). As shown in Figure 2, the male to female ratio is 1:1 and all patients including our patient presented with psychomotor delay, generalized muscle weakness and hypotonia. Almost half of them had poor head control (three patients and our index case), hyperreflexia (usually in the early phase) and hypo- or areflexia (usually late in the disease). Less common neurological symptoms described included psychomotor regression, facial dysmorphism, and microcephaly, were seen in our patient. Feeding difficulties, failure to thrive, and respiratory insufficiency were seen in almost half of the patients including our patient. Even though in this type of PCH there is motor neuron involvement, tongue fasciculation was noted in one patient. MRI showed cerebellar atrophy in almost all cases (9/9) and vermis atrophy was reported in six patients. Of note, a longitudinal MRI study performed in our patient at 2 weeks, 3 months and 1 year 8 months demonstrated the progressive course of cerebral atrophy, cerebellar atrophy, and white matter atrophy in the supratentorial area and demyelination (Figure 1). EMG showed motor neuronopathy. Three of the reported patients died at 10 years (patient 7), 2 years (patient 10, this study) and 1.5 years (patient 2) (Figure 2 and Supplementary Table S1).

This case extends the clinical, radiological, and molecular knowledge of EXOSC9 deficiency. RNA degradation and processing is a critical metabolic pathway. Severe impairments have very serious clinical consequences and, importantly, moderate alterations still have metabolic impacts. These mild defects can therefore participate in the pathogenesis of complex diseases. For example, EXOSC9 depletion has been linked to altered growth and survival in cancer cells [9]. It has been shown that a decrease in EXOSC9 leads to an increase in the level of p53 transcripts [5]. It is therefore important to identify and explore severe impairments that represent an optimal model to better understand the mechanisms underlying complex diseases.

## Figures and Tables

**Table 1:** Age of onset and molecular findings overview.

Patients	1	2	3	4	5	6	7	8	9	10
Age at onset (months)	8	Birth	Birth	0.5	36	10	11	15	2	10
Homozygous (H)/compound heterozygous (CH)	H	CH	H	H	H	H	CH	CH	H	CH
NM_001034194.1 *EXOSC9* variant 1 cDNA	c.41T>C	c.41T>C	c.41T>C	c.41T>C	c.41T>C	c.41T>C	c.239T>G	c.239T>G	c.151G>C	c.41T>C
EXOSC9 variant 1 protein	p.Leu14Pro	p.Leu14Pro	p.Leu14Pro	p.Leu14Pro	p.Leu14Pro	p.Leu14Pro	p.Leu80Arg	p.Leu80Arg	p.Gly51Arg	p.Leu14Pro
NM_001034194.1 *EXOSC9* variant 2 cDNA	c.41T>C	c.481C>T	c.41T>C	c.41T>C	c.41T>C	c.41T>C	c.484dupA	c.484dupA	c.151G>C	c.643C>T
EXOSC9 variant 2 protein	p.Leu14Pro	p.Arg161*	p.Leu14Pro	p.Leu14Pro	p.Leu14Pro	p.Leu14Pro	p.Arg162Lysfs*3	p.Arg162Lysfs*3	p.Gly51Arg	p.Arg212*
References	Burns et al. (2018) [8]	Burns et al. (2018) [8]	Burns et al. (2018) [8]	Burns et al. (2018) [8]	S. Bizzari et al. 2020 [4]	S. Bizzari et al. 2020 [4]	M. Sakamoto et al. 2021 [6]	M. Sakamoto et al. 2021 [6]	M. Sakamoto et al. 2021 [6]	This study

## Data Availability

All data are presented in the manuscript and/or in the Supplementary Materials.

## Supplementary Materials

The following supporting information can be downloaded at: https://www.mdpi.com/article/10.3390/jcm11154335/s1, Table S1: Review of the cases with EXOSC9-PHD type 1D.
